# Atypical bacterial respiratory infections in children

**DOI:** 10.36416/1806-3756/e20240126

**Published:** 2024-05-08

**Authors:** Paula Barros de Barros, Luiza Fernandes Xavier, Eduardo da Costa Herter, Maria Fernanda Gonçalves Meirelles Fernandes, Isabel Cristina Schütz Ferreira, Leonardo Araujo Pinto

**Affiliations:** 1. Centro Infant, Escola de Medicina, Pontifícia Universidade Católica do Rio Grande do Sul, Porto Alegre (RS) Brasil.; 2. Programa de Pós-Graduação em Medicina - Pediatria, Escola de Medicina, Pontifícia Universidade Católica do Rio Grande do Sul, Porto Alegre (RS) Brasil.

## INTRODUCTION AND EPIDEMIOLOGY

*Mycoplasma pneumoniae* and *Chlamydia pneumoniae* commonly cause mild infections of the respiratory system. *M. pneumoniae* infections are most common in young adults and school-aged children, but they can affect anyone. The number of *M. pneumoniae* infections varies over time, with peaks of disease every 3 to 7 years. *M. pneumoniae* infections can happen any time of the year. However, they may be more common in summer and early fall. The most common manifestation of *M. pneumoniae* infection is tracheobronchitis (chest cold). Common symptoms of a chest cold include sore throat, tiredness, fever, and slowly worsening persistent cough. *C. pneumoniae* infection can cause sore throat and ear or sinus infection. *C. pneumoniae* can also cause lower respiratory tract infections such as bronchitis. Sometimes these bacteria can cause more serious lung infections that require care in a hospital. Pneumonias caused by atypical pathogens are common etiologies and make diagnosis challenging due to their nonspecific radiological and clinical presentation. Atypical germs have been responsible for up to 23% of pneumonias in children. Clinical manifestations of atypical respiratory infections may be subacute, characterized by constitutional symptoms, with the possibility of overlapping with typical signs of conventional bacterial infections (e.g., *Streptococcus pneumoniae*). This article aims to discuss the diagnostic criteria, management, prognosis, and preventive measures related to atypical respiratory infections in children (Chart 1).^1^ ^,^ ^2^

**Chart 1:** Atypical respiratory infections.

Etiologic Agent	Respiratory Manifestations	Clinical Presentations	Diagnosis	Treatment
*Mycoplasma pneumoniae*	Pneumonia Acute Bronchitis Upper Respiratory Tract Infection Asthma Exacerbation	Insidious onset Constitutional symptoms Non-productive cough Low-grade fever Children > 3-5 years old No response to usual treatment Perihilar and bilateral pulmonary infiltrate with wheezing	Detailed medical history PCR test or serology Chest X-ray	broad-spectrum macrolides Azithromycin orally for 5 days Alternatives: Clarithromycin, Erythromycin Parenteral treatment, if necessary, with Azithromycin, Erythromycin, or Levofloxacin (> 6 months of age)
*Chlamydia pneumoniae*	Pneumonia Rhinitis Sinusitis Pharyngitis Laryngitis Acute Bronchitis		Detailed medical history PCR test or serology Chest X-ray	

## DIAGNOSIS

The diagnosis of atypical respiratory infections in children primarily relies on clinical history, age, and response to initial empirical treatment. An insidious onset, coupled with other symptoms such as headache, malaise, nonproductive cough, and low-grade fever, often suggests infections by atypical bacteria. However, despite multiple clinical presentations described in the literature, signs, symptoms, and radiological findings lack sufficient precision to differentiate atypical agents from other etiologies.^1^ ^,^ ^3^

Regarding laboratory evaluation, testing for *M. pneumoniae* or *C*. *pneumoniae* is indicated only in severely ill hospitalized children. Nasopharyngeal samples can be obtained, with tracheal aspirates being an option in intubated patients. When confirmation of *M. pneumoniae* or *C. pneumoniae* infection is necessary, PCR-based assays are preferable. PCR-based assays have high sensitivity and specificity, but should be correlated with clinical findings as they do not differentiate acute from previous recent infection. Culture may be an alternative for etiological identification but is rarely used due to the delay in obtaining results. Serological testing has commercially available kits but lacks specificity.^4^

For the confirmation of *M. pneumoniae* or *C. pneumoniae* infection, it is necessary to detect the microorganism or a specific antibody response, along with a compatible clinical syndrome. Confirmation can be achieved through convalescent serology or clinical improvement with specific therapy. Laboratory tests are indicated only if they impact patient management, especially in severe cases or if antimicrobial therapy does not cover atypical germs. In hospitalized patients with community-acquired pneumonia, especially those immunocompromised or presenting with risk factors or extrapulmonary manifestations, testing for *M. pneumoniae* and *C. pneumoniae* may be recommended. In summary, PCR assays in respiratory samples are preferred, but serology is a reasonable alternative if PCR is not available.^5^ ^)^ *C. pneumoniae* can also cause chronic infection. Some experts suggest that chronic *C. pneumoniae* infection might contribute to chronic conditions, such as difficult-to-treat or severe asthma.

## RECOMMENDED MANAGEMENT

The initial empirical treatment of pneumonia should be initiated considering the patient’s age and clinical presentation, as the pathogen is usually unknown at the time of initial diagnosis.^3^ ^)^ Furthermore, for children older than 3-5 years of age with inadequate response to usual treatment for typical pneumonia agents, particularly if there is bilateral pulmonary infiltrate, along with wheezing, testing for atypical agents may be considered, and empirical treatment against these agents may be added.^6^

In cases of suspected or confirmed infection with *C. pneumoniae* or *M. pneumoniae*, the first choice for oral treatment is azithromycin for 5 days. Azithromycin is the preferred option for these agents due to its high efficacy, prolonged half life, and low incidence of adverse effects. Other options include clarithromycin and levofloxacin. If parenteral treatment is necessary, the first option is intravenous macrolides, levofloxacin also being an option.^6^

*C. pneumoniae* is resistant to trimethoprim, sulfonamides, aminoglycosides, and glycopeptides. Penicillins have shown in vitro activity against *Chlamydia* spp., but are not recommended.^1^ ^)^ Resistance to macrolides emerged in *M. pneumoniae* and has been increasing since the 2000s. Current data suggest that the overall global prevalence of macrolide resistance in *M. pneumoniae* may be around 28%.^4^ However, there is significant geographical variation.

## PREVENTION AND PROGNOSIS

*C. pneumoniae* and *M. pneumoniae* are transmitted through person-to-person contact or via fomites. Thus, frequent handwashing, use of personal protective masks, and avoidance of contacts with symptomatic individuals are recommended for prevention. For patients hospitalized for atypical germ pneumonia or pneumonia of unknown origin, standard precautions are indicated.

In summary, pneumonia caused by atypical pathogens is typically mild and has long-lasting symptoms. Patients frequently recover during treatment without complications. In some children infected with *C. pneumoniae*, there may be prolonged cough, with an average duration of 25-30 days. The vast majority of children treated for pneumonia due to *M. pneumoniae* or *C. pneumoniae* have an excellent prognosis and fully recover.^1^
